# MYC and the unfolded protein response in cancer: synthetic lethal partners in crime?

**DOI:** 10.15252/emmm.201911845

**Published:** 2020-04-20

**Authors:** Tingting Zhang, Ningning Li, Chaoyang Sun, Yang Jin, Xia Sheng

**Affiliations:** ^1^ Ministry of Education Key Lab of Environment and Health School of Public Health Tongji Medical College Huazhong University of Science and Technology Wuhan China; ^2^ The Seventh Affiliated Hospital of Sun Yat‐sen University Shenzhen China; ^3^ Department of Obstetrics and Gynecology Tongji Hospital Tongji Medical College Huazhong University of Science and Technology Wuhan China; ^4^ Institute for Cancer Genetics and Informatics Oslo University Hospital Oslo Norway

**Keywords:** cancer, ER stress, MYC, synthetic lethality, UPR, Cancer

## Abstract

The transcription factors of the MYC family play pivotal roles in the initiation and progression of human cancers. High oncogenic level of MYC invades low‐affinity sites and enhancer sequences, which subsequently alters the transcriptome, causes metabolic imbalance, and induces stress response. The endoplasmic reticulum (ER) not only plays a central role in maintaining proteostasis, but also contributes to other key biological processes, including Ca^2+^ metabolism and the synthesis of lipids and glucose. Stress conditions, such as shortage in glucose or oxygen and disruption of Ca^2+^ homeostasis, may perturb proteostasis and induce the unfolded protein response (UPR), which either restores homeostasis or triggers cell death. Crucial roles of ER stress and UPR signaling have been implicated in various cancers, from oncogenesis to treatment response. Here, we summarize the current knowledge on the interaction between MYC and UPR signaling, and its contribution to cancer development. We also discuss the potential of targeting key UPR signaling nodes as novel synthetic lethal strategies in MYC‐driven cancers.

## Graphical abstract

This article reviews the current knowledge on the interactions between endoplasmic reticulum stress, unfolded protein response (UPR), and MYC family, and discusses recent publications that highlight the therapeutic potential of targeting key UPR signaling as a novel strategy in MYC‐driven cancers.

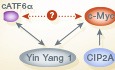

**Glossary**
Apoptosis: Controlled cell death that occurs in response to a variety of cellular stressors and as part of developmental programs of multicellular organisms.
Autophagy: Regulated mechanism used by the cells to maintain homeostasis and normal function through orderly degradation and recycling of unnecessary or dysfunctional components.
Autophosphorylation: Phosphorylation of the protein kinase by itself, which plays an important role in the process of cell signal transduction.
Dimerization: Chemical reaction that binds two molecular subunits, resulting in the formation of a single dimer.
ER stress (Endoplasmic reticulum stress): Stress caused by the accumulation of misfolded and unfolded proteins in the ER lumen or by Ca^2+^ balance disorders.
ERAD (Endoplasmic reticulum‐associated degradation): Umbrella term that covers a range of different mechanisms by which misfolded proteins are retained in the ER and delivered for proteasomal degradation after retrotranslocation into the cytosol.
GEMM (Genetically engineered mouse model): Mouse model for research on human diseases, in which the mouse genome is altered through the use of genetic engineering techniques.
Gluconeogenesis: Metabolic process in which glucose is formed from non‐carbohydrate precursors.
Metabolic reprogramming: Molecular adjustments in metabolic pathways that alter the bioenergetic profile and metabolism of the cell.
PDX (Patient‐derived xenograft): Mouse model based on transplantation and serial propagation of fresh human tumor biopsies in immunodeficient mice.
Proteostasis: Homeostatic mechanisms controlling the biogenesis, trafficking, and degradation of proteins in cells. Its imbalances may lead to the aggregation of misfolded proteins, trigger stress responses, or excessive protein degradation.
RIDD (Regulated IRE1α‐dependent decay): Degradation of mRNAs encoding mostly ER‐targeted proteins by IRE1α, to reduce the load of incoming ER “client” proteins during ER stress.
Tumor microenvironment: Cellular environment in which tumor cells reside. It consists of extracellular matrix and different populations of stromal cells, including endothelial cells, fibroblasts, and immune cells.
UPR (Unfolded protein response): Collection of phylogenetically conserved signaling pathways initiated by transmembrane stress sensors of the endoplasmic reticulum.

## The MYC family and cancer

*MYC* gene encodes the basic helix–loop–helix/leucine zipper (bHLH‐LZ) transcription factor c‐Myc that belongs to the MYC family, together with L‐Myc and N‐Myc (encoded by *MYCL* and *MYCN,* respectively). These genes are differentially expressed during development, but the MYC proteins are functionally equivalent in most biological systems (Conacci‐Sorrell *et al*, 2014). c‐Myc heterodimerizes with MAX, another bHLH‐LZ protein, and the complex binds DNA sequences enriched in the promoters and enhancers to regulate gene expression. The canonical high‐affinity sites of c‐Myc‐MAX heterodimer are termed “E‐boxes” with a consensus sequence 5′‐CACGTG‐3′ (Blackwell *et al*, 1993; Fernandez *et al*, 2003). In malignant cells where c‐Myc expression exceeds normal level, c‐Myc can bind DNA sequences beyond E‐boxes (Wolf *et al*, 2015). Upon DNA binding, c‐Myc‐MAX recruits the positive transcription elongation factor complex, which subsequently phosphorylates RNA polymerase II to increase transcription rate (Rahl *et al*, 2010). In addition to its well‐established role as a transcriptional activator, c‐Myc can also repress expression of numerous target genes when transcriptional co‐repressors are recruited to the c‐Myc‐MAX complex (Kleine‐Kohlbrecher *et al*, 2006).

As a global transcriptional regulator, c‐Myc can bind to approximately 10‐15% of the genome and regulate the expression of both protein‐encoding genes and non‐coding RNAs, which have been implicated in various cellular processes such as proliferation, growth, apoptosis, energy metabolism, and diverse biosynthetic pathways (Kress *et al*, 2015; Hsieh & Dang, 2016). By acting on RNA polymerases, c‐Myc not only upregulates target gene expression, but also promotes the synthesis of rRNA and tRNA, thus stimulating both transcription and translation of various ribosomal proteins and eukaryotic translation initiation factors. c‐Myc thereby activates the entire protein synthetic apparatus required for cancer cell growth (Dunn & Cowling, 2015; Stine & Dang, 2015). Furthermore, c‐Myc reprograms the metabolic landscape to generate building blocks (such as amino acids and lipids) essential for increased biomass and growth of cancer cells (Stine *et al*, 2015).

Alterations in *MYC* oncogene are a hallmark of many human cancers (Beroukhim *et al*, 2010). Constitutive c‐Myc activation can result from diverse mechanisms, such as chromosomal translocation and rearrangements, which frequently occur in Burkitt's lymphoma and multiple myeloma (Dalla‐Favera *et al*, 1982; Shou *et al*, 2000). In tumors where *MYC* is not amplified, loss of the tumor suppressor adenomatous polyposis coli and activation of the WNT/β‐catenin pathway lead to transcriptional activation of *MYC* via TCF transcription factor, a phenomenon occasionally observed in colorectal and prostate cancers (He *et al*, 1998; Nandana & Chung, 2014). While wild‐type c‐Myc has a half‐life of 15‐20 min, mutations in c‐Myc residues (such as Thr58 and Ser62) increase protein stability and contribute to *in vivo* tumorigenesis (Wang *et al*, 2011). c‐Myc overexpression is observed in up to 70% viral and alcohol‐related hepatocellular carcinoma and is associated with an aggressive phenotype (Schlaeger *et al*, 2008; Lin *et al*, 2010). Similarly, *MYCN* is frequently deregulated in solid tumors of neuroendocrine and neuronal origin. In neuroblastoma, the most common extracranial pediatric solid tumor, *MYCN* amplification is an important clinical biomarker associated with poor prognosis (Grimmer & Weiss, 2006). Furthermore, N‐Myc is a critical driver of neuroendocrine prostate cancer, a subtype of castration‐resistant prostate cancer with neuroendocrine features (Wyatt & Gleave, 2015; Dardenne *et al*, 2016). Finally, L‐Myc is the least understood member of this oncoprotein family, with a much lower transforming capacity than c‐Myc or N‐Myc (Birrer *et al*, 1988; Barrett *et al*, 1992). However, *MYCL* amplification is detected in small‐cell lung cancer more frequently than *MYC* or *MYCN* amplification and is believed to play a tumorigenic role therein (Kim *et al*, 2016).

## ER stress and UPR signaling

**Figure 1:**
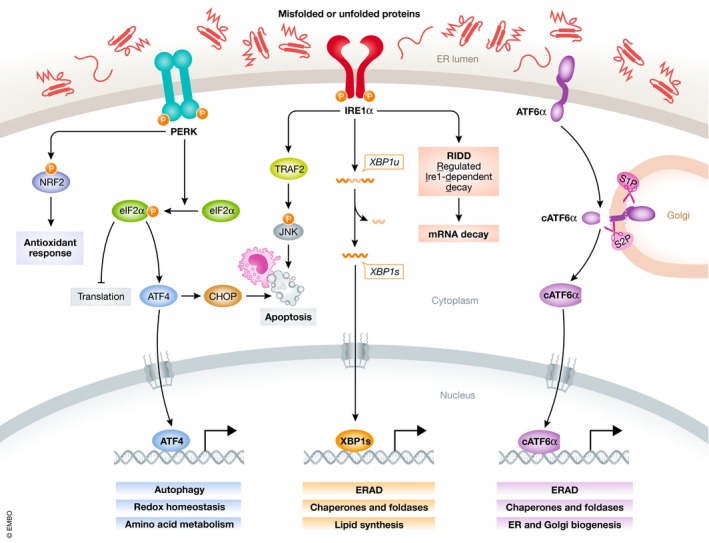
The three arms of the UPR pathways Accumulation of misfolded or unfolded proteins in the ER lumen activates the three UPR pathways initiated by PERK, IRE1α, and ATF6α. This leads to either the recovery of ER homeostasis by blocking protein translation and enhancing protein‐folding capacity and clearance of misfolded proteins, or apoptosis upon unresolved ER stress.

The endoplasmic reticulum (ER) contributes to the proper functioning of the secretory pathway by providing a complex network of chaperones, foldases, cofactors, and quality control mechanisms (Wang & Kaufman, 2014). It is also involved in metabolic processes including lipid synthesis, gluconeogenesis, and calcium metabolism (Schwarz & Blower, 2016). Perturbations in ER homeostasis, such as disrupted proteostasis, lead to accumulation of misfolded or unfolded proteins in the ER lumen. This stress triggers an adaptive mechanism named the unfolded protein response (UPR), which increases ER chaperone expression, improves the clearance of misfolded proteins via ER‐associated degradation (ERAD), and attenuates protein translation (Walter & Ron, 2011; Ruggiano *et al*, 2014; Hetz *et al*, 2015). On the other hand, the UPR initiates apoptotic signaling when the damage is irremediable (Kim *et al*, 2008). The canonical UPR is initiated by three ER transmembrane stress sensors: inositol‐requiring enzyme 1 (IRE1, IRE1α, and IRE1β), protein kinase R‐like ER kinase (PERK), and activating transcription factor 6 (ATF6, ATF6α, and ATF6β) (Fig 1). They are maintained inactive when their luminal domains are bound to the glucose‐regulated protein (GRP) 78 (Hotamisligil, 2010; Walter & Ron, 2011).

IRE1α comprises a kinase domain and an endoribonuclease domain on its cytosolic region. In response to the accumulation of unfolded or misfolded proteins in the ER lumen, IRE1α undergoes dimerization and trans‐autophosphorylation. This conformational change activates its RNase domain, which excises a 26‐nucleotide intron within the *XBP1* mRNA (Yoshida *et al*, 2001; Calfon *et al*, 2002). This results in the expression of spliced XBP1 (XBP1s), a potent transcription factor that regulates numerous genes involved in protein folding, quality control, ERAD, and lipid synthesis (Karagoz *et al*, 2019). Under certain conditions, IRE1α also cleaves mRNAs, rRNAs, and miRNAs through its RNase domain via regulated IRE1α‐dependent decay (RIDD), which either preserves ER homeostasis or facilitates cell death (Hollien *et al*, 2009; Coelho & Domingos, 2014). When faced with unresolved stress, IRE1α may induce apoptosis by activating the c‐Jun N‐terminal kinase (JNK) signaling (Urano *et al*, 2000; Dhanasekaran & Reddy, 2008).

Once dissociated from GRP78, PERK undergoes dimerization and autophosphorylation, which activates its cytosolic kinase domain and phosphorylates Ser51 in eukaryotic translation initiation factor 2 (eIF2) α‐subunit (Liu *et al*, 2000; Holcik & Sonenberg, 2005). This transiently halts global translation and decreases the load of nascent proteins entering the ER (Wang & Kaufman, 2016). Meanwhile, it allows translation of a small subset of mRNAs with specific upstream open reading frames, such as *ATF4* (Harding *et al*, 2000a). ATF4 is a key transcription factor that promotes adaptive response by regulating the expression of genes involved in protein folding, autophagy, and redox homeostasis (Wortel *et al*, 2017). It also transactivates the pro‐apoptotic protein C/EBP homologous protein (CHOP) under chronic ER stress and triggers apoptosis (Averous *et al*, 2004). Three additional kinases, protein kinase R, heme‐regulated eIF2α kinase, and general control nonderepressible 2 (GCN2), phosphorylate eIF2α at the same residue, which are collectively known as the “integrated stress response” (Pakos‐Zebrucka *et al*, 2016).

ATF6α translocates to the Golgi apparatus upon ER stress, where it is proteolytically processed by the site‐1 and site‐2 proteases (S1P and S2P), generating a cytosolic fragment that functions as a basic leucine zipper transcription factor (Haze *et al*, 1999). ATF6α transcriptionally upregulates the expression of many ER chaperones, as well as key UPR component genes such as *XBP1* (Yoshida *et al*, 2001; Shoulders *et al*, 2013). It also plays a role in ERAD, for instance by forming heterodimers with XBP1s, and drives specific gene expression programs (Yamamoto *et al*, 2007).

## UPR signaling in cancer

The UPR is often co‐opted by cancer cells to cope with the increased protein synthesis or the hostile tumor microenvironment (such as hypoxia and nutrient deprivation). Recently, several studies have provided comprehensive insights on the role of UPR in promoting different cancers (Clarke *et al*, 2014; Chevet *et al*, 2015; Storm *et al*, 2016; Urra *et al*, 2016; Madden *et al*, 2019; Wang *et al*, 2019).

For example, the IRE1α‐XBP1s arm helps triple‐negative breast cancer cells (TNBC) overcome hypoxic conditions by interacting with HIF1α and cooperatively regulating its transcriptional network (Chen *et al*, 2014). IRE1α‐XBP1s is also directly activated by androgen receptor signaling in prostate cancer cells and promotes their survival (Sheng *et al*, 2015). XBP1s rewires key metabolic pathways, which enables cancer cells to survive nutrient shortage conditions via transcriptional regulation of several rate‐limiting enzymes involved in hexosamine biosynthesis (Wang *et al*, 2014; Madden *et al*, 2019). In glioblastoma, *XBP1* splicing promotes tumor stroma remodeling, angiogenesis, and invasion, whereas IRE1α‐mediated RIDD for miR‐17 displays anti‐angiogenic and antimigratory effects, suggesting a dual role of IRE1 RNase in glioblastoma aggressiveness (Lhomond *et al*, 2018). The function of PERK is also dependent on the context. PERK and eIF2α phosphorylation is suppressed in proliferative prostate cancer cells stimulated by androgens (Sheng *et al*, 2015), whereas ATF4 is essential for prostate cancer growth and survival (Pallmann *et al*, 2019). PERK activation is also shown to confer hypoxia tolerance and radiotherapy resistance to different tumor cells by upregulating expression of autophagy‐related genes via ATF4 and CHOP (Rouschop *et al*, 2010). Pharmacological inhibition of PERK kinase activity triggers robust antitumor effect in multiple preclinical models of pancreatic cancer and multiple myeloma (Atkins *et al*, 2013). ATF6α also appears to play a cytoprotective role, such as in *TP53* mutant tumor cells (Sicari *et al*, 2019). It is required for tumor cell dormancy and contributes to resistance to chemotherapy and radiotherapy by activating mTOR and NOTCH signaling, respectively (Schewe & Aguirre‐Ghiso, 2008; Dadey *et al*, 2016). Additional critical functions of UPR signaling consist of reshaping the tumor stroma (Tyekucheva *et al*, 2017), especially that of cancer‐associated immune cells (Cubillos‐Ruiz *et al*, 2017). For instance, persistent activation of the IRE1α‐XBP1s axis in tumor‐associated dendritic cells and T cells disrupts their metabolic homeostasis, which results in impaired immunosuppression in ovarian cancer models (Cubillos‐Ruiz *et al*, 2015, 2017; Song *et al*, 2018).

Therefore, and contrary to what was originally thought, UPR signaling in cancer cells has a profound and complex impact on tumor initiation, progression, metastasis, and tumor microenvironment (Clarke *et al*, 2014; Dufey *et al*, 2015). Over the last few years, small molecules modulating the activity of specific UPR branches or components have been developed, and some of them are currently under clinical evaluation (Hetz *et al*, 2013, 2019; Jin & Saatcioglu, 2020). In line with this effort, identification of cancers potentially responsive to drugs targeting the UPR will be of great importance.

## Interaction between MYC and UPR in cancer

During tumor development, protein synthesis rate is tightly regulated to sustain cell survival. Increased protein synthesis requires concomitant increased folding capacity to avoid proteotoxicity (Harding *et al*, 2000b). MYC activation constitutes an intrinsic stress that places further weight on protein synthesis and secretion (Tameire *et al*, 2015). While the ER constitutes a link between these intracellular processes and the changes in cellular biomass and growth, it has been underappreciated in the context of MYC‐hyperactivated cancers until recently.

We summarize below the direct and indirect connections found between MYC and UPR activation in different cancers and propose that MYC and UPR activation may work together to foster tumor progression. We also discuss the therapeutic potential of targeting UPR signaling in cancers with MYC overexpression.

## Indirect regulation of UPR by MYC

Remarkably, UPR is induced in tumors with MYC alterations. For example, PERK‐eIF2α pathway is selectively activated in a mouse model of prostate cancer with *MYC* hyperactivation and is believed to hijack global protein synthesis required for cancer progression (Nguyen *et al*, 2018). Similarly, c‐Myc‐enhanced protein synthesis induces an adaptive ER stress response in mice with malignant rhabdoid tumors of the liver, while c‐Myc depletion decreases the levels of GRP78, ATF4, and CHOP (Carugo *et al*, 2019).

As a vital piece of the proteostasis system, autophagy is frequently activated to clear misfolded proteins following MYC‐induced proteotoxicity (Levy *et al*, 2017). In lymphoma cells, both c‐Myc and N‐Myc activate PERK‐eIF2α‐ATF4 signaling, which induces cytoprotective autophagy and attenuates ER Ca^2+^ release to support malignant transformation and survival (Hart *et al*, 2012). In *Drosophila*, Myc induces autophagy and cell overgrowth by activating another PERK effector, nuclear factor erythroid 2‐related factor 2 (Nrf2), a master transcription factor mediating the antioxidant responses (Cullinan *et al*, 2003; Ma, 2013; Nagy *et al*, 2013).

Furthermore, MYC direct targets also contribute to the regulation of ER stress and autophagy. As an example, N‐myc downstream‐regulated gene 1 (*NDRG1*) is transcriptionally repressed by both N‐Myc and c‐Myc, and inhibits PERK‐mediated autophagic pathway (Okuda & Kondoh, 1999; Sahni *et al*, 2014). A recent study further shows that NDRG1 inhibits IRE1α arm while facilitating ATF6α cleavage and inducing the expression of GRP78, calreticulin, and calnexin (Merlot *et al*, 2019). Thus, NDRG1 provides another molecular hub linking MYC with activation of UPR and autophagy.

On the other hand, MYC may suppress autophagy to induce ER stress. In non‐small‐cell lung cancer models, c‐Myc transcriptionally activates miR‐150, which blocks the fusion of autophagosomes and lysosomes through direct inhibition of EPG5. The miR‐150‐mediated autophagy defect further induces ER stress and promotes tumor growth (Li *et al*, 2019). Bioinformatics analysis predicts that miR‐214‐3p is c‐Myc‐regulated and likely controls the expression of XBP1 in B‐cell lymphoma, yet its function remains to be determined (Malpeli *et al*, 2018).

Another link between MYC and UPR in cancer is the rewired metabolism. Elevated ATF4 expression is a common feature of neuroblastoma cells with *MYCN* amplification and is responsible for the activation of the serine–glycine synthesis pathways essential for cell survival (Locasale, 2013; Liu *et al*, 2016). MYC also alters mitochondrial metabolism in these cells, making them vulnerable to glutamine deprivation. In this context, ATF4 is activated by GCN2‐eIF2α axis and promotes apoptosis by inducing *PUMA*,* NOXA,* and *TRB3* expression (Qing *et al*, 2012). Likewise, blockade of essential amino acid transport triggers the GCN2‐eIF2α‐ATF4 pathway and inhibits neuroblastoma tumor growth, which is concomitant with attenuated translation of *MYC* and *MYCN* mRNAs (Yue *et al*, 2017). Therefore, the role of ATF4 in neuroblastoma cells with elevated MYC varies depending on the condition.

Notably, GCN2‐eIF2α‐ATF4 activation by MYC was recently described. By generating excess uncharged tRNAs, c‐Myc induces an optimal expression of ATF4. Then, c‐Myc and ATF4 cooperate to regulate a specific program of c‐Myc target genes, mainly involved in amino acid and protein synthesis (Tameire *et al*, 2019). One of these targets is eIF4E‐binding protein 1 (4E‐BP1), a repressor of eIF4F complex and mRNA translation (Gingras *et al*, 1999). Thus, these results provide additional mechanisms by which eIF2α phosphorylation regulates translation rate and maintains proteostasis in malignant cells with MYC overexpression.

In addition, both the RNase and kinase activities of IRE1α have been implicated in MYC‐hyperactivated tumors. In c‐Myc‐overexpressing endocrine‐resistant breast cancer cells, IRE1α activation turns on either JNK signaling for apoptosis or *XBP1* splicing for survival (Shajahan‐Haq *et al*, 2014). In pancreatic ductal adenocarcinoma cells with activated c‐Myc, IRE1α induces the MKK4‐JNK signaling and the ATF2 transcriptional program, driving an adaptive response to the increased protein metabolism (Genovese *et al*, 2017). In contrast, XBP1s transactivates SIRT7 in liver cancer cells, which represses translation by cooperatively inhibiting transcription of genes encoding ribosomal proteins with c‐Myc (Shin *et al*, 2013). Therefore, indirect interaction between c‐Myc and IRE1α may also mitigate proteotoxicity and ER stress.

## Direct regulation of UPR by MYC

Beside indirect regulation, recent studies have also shed light on the direct regulation of UPR by MYC. Zhao and colleagues have shown that c‐Myc is required for the activation of the IRE1α‐XBP1s pathway in TNBC models: Genetic knockdown of c‐Myc leads to a marked decrease in IRE1α and XBP1s, rescued by ectopic expression of c‐Myc. Chromatin immunoprecipitation (ChIP) and luciferase reporter assays further demonstrate that c‐Myc transactivates *ERN1* gene expression by directly binding to multiple sites in its proximal promoter and enhancer (Zhao *et al*, 2018). Along these lines, another study in Burkitt's lymphoma cells reports that c‐Myc binds the E‐box sequences in the promoters of both *ERN1* and *XBP1* genes (Xie *et al*, 2018), establishing c‐Myc as a direct upstream regulator of the IRE1α‐XBP1s pathway.

At the protein level, c‐Myc physically interacts with XBP1s and enhances its transcriptional activity in TNBC models (Zhao *et al*, 2018). Furthermore, while the mechanism remains unknown, c‐Myc is crucial for IRE1α protein stability in Burkitt's lymphoma cells (Xie *et al*, 2018). As an example, IRE1α‐XBP1s mediates the oncogenic effect of c‐Myc by upregulating the expression of stearoyl‐CoA desaturase 1 (SCD1), which generates unsaturated lipids to maintain ER membrane homeostasis despite c‐Myc‐dependent proteotoxicity (Xie *et al*, 2018).

Several recent studies have also shed light on the direct regulation of PERK pathway components by MYC. c‐Myc binds and activates *ATF4* promoter, which plays a role in anoikis resistance in human osteosarcoma cells (Mo *et al*, 2018) and in response to bortezomib in Elt3 rat leiomyoma cells (Babcock *et al*, 2013). Similarly, N‐Myc and ATF4 collectively drive the metabolic reprogramming in neuroblastoma cells, leading to dependency on the serine–glycine–one‐carbon metabolic pathway. Mechanistically, N‐Myc transactivates *ATF4* expression while ATF4 contributes to the stabilization of N‐Myc protein by antagonizing its ubiquitination in a positive feedback loop (Xia *et al*, 2019). In addition, ATF3, an ATF4 target with critical functions in cell fate determination under stress conditions, is also directly regulated by c‐Myc and plays a role in mediating its proliferative effect (Tamura *et al*, 2005). c‐Myc‐mediated transcriptional repression plays a critical role in preventing cells from exiting cell cycle and in facilitating proliferation via inhibition of growth arrest and DNA damage (GADD) gene expression, such as *GADD153* that encodes CHOP (Chen *et al*, 1996; Amundson *et al*, 1998). c‐Myc‐MAX complex binds to the minimal promoter region of *GADD153 in vivo*, where it prevents transcriptional activator c‐Myc‐interacting zinc finger protein 1 (Miz‐1) activity and impairs gene expression (Barsyte‐Lovejoy *et al*, 2004; Wiese *et al*, 2013). Taken together, these studies suggest that UPR signaling is tightly regulated by MYC and plays a key role in mediating its oncogenic effect.

## Indirect regulation of MYC by UPR

Importantly, the link between MYC and UPR does not appear to be only one way, as ER stress signaling has also been shown to affect MYC expression. The calcium‐dependent serine/threonine phosphatase calcineurin is activated upon disruption in calcium homeostasis and ER stress, and activates a number of transcription factors, one of them being the nuclear factor of activated T cell (NFAT) (Bonilla *et al*, 2002). Activated NFAT directly binds to the proximal *MYC* promoter and stimulates its transcription, ultimately resulting in enhanced anchorage‐dependent and anchorage‐independent growth of pancreatic cancer cells (Buchholz *et al*, 2006). In multiple myeloma cells, c‐Myc protein level is maintained despite global decreased protein synthesis mediated by PERK‐eIF2α activation, owing to the upregulated activity of the *MYC* mRNA internal ribosome entry site upon ER stress (Shi *et al*, 2016).

## Direct regulation of MYC by UPR

One of the most intriguing findings is that XBP1s also directly regulates MYC expression. Exogenous XBP1s has previously been shown to dose‐dependently enhance the reporter activity driven by *MYC* promoter (Chae *et al*, 2016). A similar phenomenon is also observed in colon cancer cells co‐transfected with XBP1s expression vector and *MYC* luciferase reporter. This is significantly reversed when Fbw7, a substrate recognition component of the SKP1‐Cullin‐F‐box‐type E3 ligase, is introduced, as Fbw7 interacts with XBP1 and facilitates its ubiquitination and degradation (Chae *et al*, 2019).

**Figure 2:**
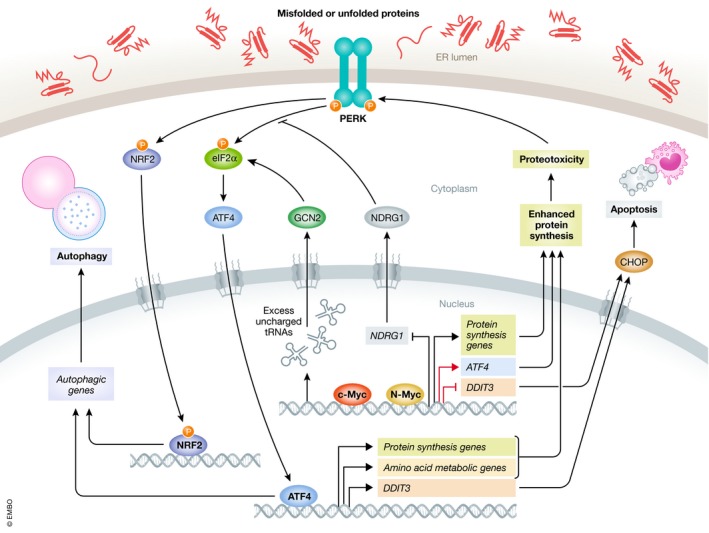
Interaction between MYC and PERK pathway Oncogenic MYC upregulates the expression of genes involved in protein synthesis, such as ATF4, which may result in proteotoxicity. PERK‐eIF2α‐ATF4 pathway is often activated upon this intrinsic stress, which subsequently induces cytoprotective autophagy. Alternatively, PERK may activate autophagy by phosphorylating NRF2. Meanwhile, GCN2‐eIF2α‐ATF4 axis can be activated by c‐Myc‐induced excess tRNAs, resulting in metabolic reprogramming and enhanced protein synthesis. In addition, MYC mediates transcriptional repression on *NDRG1* and *DDIT3*, which leads to enhanced cytoprotective autophagy and suppressed apoptosis, respectively. Red arrows indicate direct transcriptional regulation of PERK arm by MYC.

**Figure 3:**
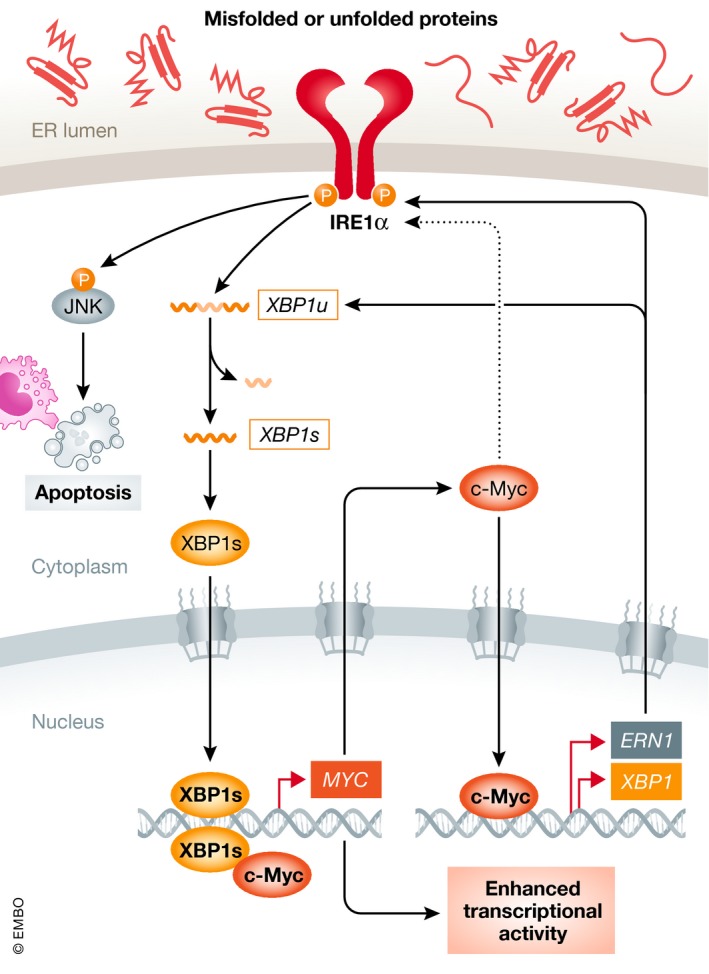
The positive feedback loop between MYC and IRE1α pathway In MYC‐hyperactivated tumors, IRE1α‐XBP1s signaling and MYC likely engage in a positive feedback loop, as XBP1s transcriptionally upregulates *MYC* while c‐Myc directly induces *ERN1* (encoding IRE1α) and *XBP1* expression. c‐Myc also physically interacts with XBP1s and enhances its transcriptional activity. MYC is further shown to contribute to IRE1α protein stability via unknown mechanisms (denoted as dashed line). Red arrows indicate direct transcriptional regulation between MYC and IRE1α arm.

Consistently, our recent study in prostate cancer cells demonstrates that XBP1s directly transactivates *MYC* expression. Strikingly, c‐Myc and XBP1s transcriptional activities are positively correlated in multiple prostate cancer patient cohorts, underscoring the fact that these two critical transcription factors are often concurrently activated in prostate cancer (Sheng *et al*, 2019). Furthermore, a recent study shows that activities of c‐Myc and AR pathways are significantly correlated in prostate cancer, while c‐Myc depletion leads to decreased expression of full‐length AR, as well as of several AR splice variants involved in AR‐targeted therapy resistance (Bai *et al*, 2019). Therefore, androgen signaling, IRE1α‐XBP1s pathway, and c‐Myc may form a dynamic trio to support prostate cancer progression. Interestingly, this direct regulation of MYC by IRE1α‐XBP1s is not restricted to cancer cells, as a recent study reports that XBP1s also upregulates *MYC* expression to promote proliferation of natural killer cells (Dong *et al*, 2019). Taken together, these data reinforce the hypothesis of a positive feedback loop between MYC and IRE1α‐XBP1s pathway, which may be a critical driver of various MYC‐dependent cancers. The major findings on the interactions between MYC and PERK or IRE1 are summarized in Figs 2 and 3, respectively.

**Figure 4:**
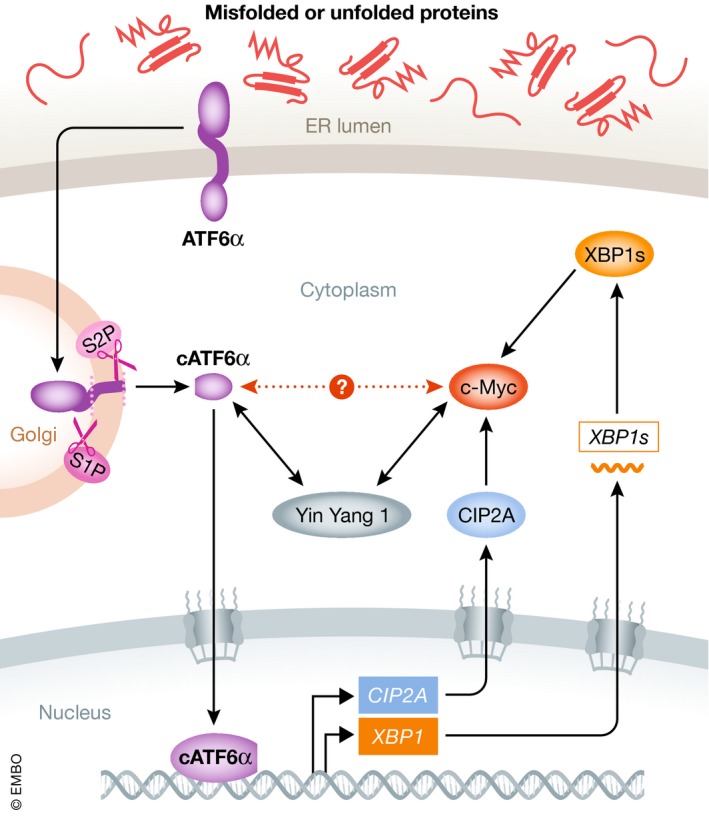
The interaction between MYC and ATF6α pathway ATF6α directly induces the expression of CIP2A, which interacts with and stabilizes c‐Myc protein. As a direct target of ATF6α, XBP1 also contributes to sustain c‐Myc expression. Meanwhile, ATF6α interactor Yin Yang 1 transcription factor has been shown to associate with c‐Myc, but whether these two proteins interact with each other is not known (denoted as red dashed line).

Comparatively, much less is known about the interaction between MYC and ATF6α in malignant conditions. Indirect evidence suggests that ATF6α promotes MYC activity. Indeed, ATF6α transcriptionally induces the expression of cancerous inhibitor of PP2A (CIP2A), which directly interacts with and stabilizes c‐Myc protein (Liu *et al*, 2018). ATF6α also induces XBP1 expression, which is capable of activating c‐Myc expression (Yoshida *et al*, 2001; Sheng *et al*, 2019). Furthermore, protein–protein interaction databases (such as BioGRID) indicate that the known ATF6α interactor Yin Yang 1 transcription factor associates with c‐Myc (Shrivastava *et al*, 1993; Li *et al*, 2000). Thus, it is worth investigating whether these proteins form a complex, and what would then be its functional significance (Fig 4). Lastly, it is reasonable to speculate that ATF6α‐mediated elevation in chaperone expression and ERAD is required for coping with the increased nascent protein load driven by MYC.

Interestingly, a direct connection between MYC and ERAD was recently established, as c‐Myc activates ubiquitin fusion degradation 1 (*UFD1*) to promote progression of T‐cell acute lymphoblastic leukemia (Huiting *et al*, 2018). UFD1 is an E2 component of the ERAD complex and facilitates the elimination of misfolded proteins from the ER, whereas *UFD1* knockdown exacerbates ER stress, activates PERK‐CHOP pathway, and induces apoptosis (Wolf & Stolz, 2012; Huiting *et al*, 2018). Nevertheless, the potential crosstalk between MYC and ATF6α signaling as well as ERAD remains to be explored.

## Targeting UPR in MYC‐driven cancers

Building upon these critical findings, targeting the UPR has been proposed as a novel therapeutic strategy in tumors with hyperactivated MYC. Here, we highlight the application and efficacy of targeting UPR signaling in MYC‐hyperactivated cancers (Table 1).

**Table 1:** Strategies and outcomes of targeting UPR in MYC‐hyperactivated cancer models

UPR branch	Compound/Intervention	Target	Experimental models	Effect	Synergy	References
PERK	GSK2606414	PERK kinase	Multiple neuroblastoma cell lines and xenografts	Reduce autophagy and inhibit growth	With GLI inhibitor GANT‐61	Axten *et al* (2013), Wang *et al* (2018)
	ISRIB	eIF2B	PCa mouse models and PDX	Impair cancer development, prolong survival, and inhibit metastases	/	Tsai *et al* (2018), Nguyen *et al* (2018)
	Genetic depletion	*PERK*	Transformed MEFs allografted in immunodeficient mice	Inhibit growth	/	Hart *et al* (2012)
	Genetic depletion	*PERK*	*Drosophila* fat body cells	Inhibit overgrowth	/	Nagy *et al* (2013)
	Genetic depletion	*ATF4*	MEFs and lymphoma mouse models	Induce apoptosis and prolong tumor‐free and overall survival	/	Tameire *et al* (2019)
IRE1α	MKC8866	IRE1α RNase	TNBC PDX and GEMM	Inhibit growth	With docetaxel	Sanches *et al* (2014), Zhao *et al* (2018), Logue *et al* (2018)
	MKC8866	IRE1α RNase	Multiple PCa cell lines and xenografts	Inhibit growth	With cabazitaxel	Sheng *et al* (2019)
	B‐I09	IRE1α RNase	Multiple BL cell lines and xenografts	Inhibit growth and induce apoptosis	With doxorubicin or vincristine	Xie *et al* (2018)
	Genetic depletion	*ERN1*	3D PDAC cell growth and orthotopic transplants	Inhibit growth and suppress tumorigenicity	/	Genovese *et al* (2017)

BL, Burkitt's lymphoma; GEMM, genetically engineered mouse model; MEFs, mouse embryonic fibroblasts; PCa, prostate cancer; PDAC, pancreatic ductal adenocarcinoma; TNBC, triple‐negative breast cancer.

Genetic ablation of PERK significantly attenuates the growth of transformed mouse embryonic fibroblasts (MEFs) with induced c‐Myc expression allografted in immunodeficient mice (Hart *et al*, 2012). Similarly, PERK depletion prevents Myc‐induced overgrowth of fat body cell clones in *Drosophila* (Nagy *et al*, 2013). Furthermore, ATF4 ablation significantly reduces *in vitro* clonogenic survival of MEFs with high c‐Myc level and extends tumor‐free and overall survival in syngeneic mouse model of lymphoma with hyperactive c‐Myc (Tameire *et al*, 2019). PERK inhibition with an optimized kinase inhibitor, GSK2606414 (Axten *et al*, 2013), reduces autophagy in *MYCN*‐amplified neuroblastoma cells and further enhances the efficacy of GLI inhibitor in repressing the growth of these cells *in vitro* and *in vivo* (Wang *et al*, 2018). ISRIB is a small‐molecule compound that enhances the guanine nucleotide‐exchanging activity of eIF2B and its interaction with eIF2α, and thus re‐activates protein synthesis despite of eIF2α phosphorylation (Tsai *et al*, 2018). ISRIB impairs cancer development, prolongs survival of different prostate cancer mouse models, and decreases metastatic progression in an advanced castration‐resistant prostate cancer patient‐derived xenograft (PDX) model (Nguyen *et al*, 2018).

In parallel, genetic silencing of XBP1 selectively blocks the growth of c‐Myc‐hyperactivated TNBC cells. Pharmacological inhibition of IRE1α RNase activity using an optimized hydroxy‐aryl‐aldehyde compound MKC8866 counteracts the growth of c‐Myc‐overexpressing TNBC tumors in both PDX and genetically engineered mouse models (Sanches *et al*, 2014; Zhao *et al*, 2018). Similarly, pharmacological and genetic inhibition of XBP1 induce c‐Myc‐dependent apoptosis of Burkitt's lymphoma models, which is alleviated by exogenous unsaturated fatty acids (Xie *et al*, 2018). In the mesenchymal pancreatic ductal adenocarcinoma mouse models with activated c‐Myc, constitutive knockdown of *Ern1* potently impairs 3D clonogenic cell growth and suppresses tumorigenicity in orthotopic transplants *in vivo* (Genovese *et al*, 2017). Likewise, disruption of the IRE1α‐XBP1s pathway by either RNA interference or small molecules targeting IRE1α RNase results in significant repression in the growth of multiple prostate cancer xenografted tumors (Sheng *et al*, 2015, 2019).

Of note, these studies also unanimously demonstrate that IRE1α RNase inhibition augments the effect of chemotherapy, a strategy with inferior therapeutic efficacy in MYC‐high tumors (Savage *et al*, 2009; Emadali *et al*, 2013; Lee *et al*, 2017). IRE1α RNase inhibition enhances the cytotoxic effect of doxorubicin or vincristine in different c‐Myc‐overexpressing Burkitt's lymphoma cells *in vitro* (Xie *et al*, 2018). In prostate cancer xenograft models, a strong synergistic tumor growth inhibition is observed when MKC8866 treatment is combined with cabazitaxel (Sheng *et al*, 2019). In TNBC, the same IRE1α RNase inhibitor substantially enhances the efficacy of docetaxel in PDX as well as syngeneic *p53*‐null transgenic mouse models with c‐Myc hyperactivation (Zhao *et al*, 2018). These findings coincide with a recent TNBC study showing that MKC8866 increases the effectiveness of xenografted tumors to paclitaxel, which may be due to the modulation of the tumor cell secretome (Logue *et al*, 2018). Nevertheless, these data certainly underline the potential of targeting IRE1α either as a monotherapy in MYC‐high tumors or in combination with chemotherapy in the future.

## Conclusions

Direct pharmacological inhibition of MYC has proven to be challenging. Thus, alternative means, such as targeting MYC synthetic lethal partners, have raised interest. The reprogrammed growth, proliferation, and metabolism driven by oncogenic MYC render cancer cells more vulnerable to the disruption of certain biological processes on which they rely. MYC activation has been shown to be synthetically lethal with inhibition of translation, spliceosome, cell cycle, and metabolism (Stine & Dang, 2015; Hsieh & Dang, 2016). The ER stress response now takes its place among these synthetic lethal targets. However, despite exciting recent progress, further preclinical and clinical evaluation will be needed to establish rational therapeutic design. Importantly, biomarkers should also be identified to help discriminating patients that may benefit from different UPR inhibitors.

## Conflict of interest

The authors declare that they have no conflict of interest.

**Pending issues**

(i) Detailed knowledge of the interactions between MYC and the entire UPR network, depending on the context.
(ii) Elucidating the potential direct interaction between MYC and ATF6α and its functional role in different cancers and cancer phases.
(iii) In‐depth preclinical evaluation and optimization of strategies targeting the UPR in MYC‐hyperactivated cancers.
(iv) Uncovering synergy and its underlying mechanism between compounds modulating UPR activity and clinical drugs, such as chemotherapeutic agents, in MYC‐high tumors.
(v) Translation of the basic and preclinical knowledge into clinical application.
